# Spatial distribution of transcript changes in the maize primary root elongation zone at low water potential

**DOI:** 10.1186/1471-2229-8-32

**Published:** 2008-04-03

**Authors:** William G Spollen, Wenjing Tao, Babu Valliyodan, Kegui Chen, Lindsey G Hejlek, Jong-Joo Kim, Mary E LeNoble, Jinming Zhu, Hans J Bohnert, David Henderson, Daniel P Schachtman, Georgia E Davis, Gordon K Springer, Robert E Sharp, Henry T Nguyen

**Affiliations:** 1 Division of Plant Sciences, University of Missouri, Columbia, MO 65211, USA; 2 Department of Animal Science, University of Arizona, Tucson, Arizona 85721, USA; 3 Department of Computer Science, University of Missouri, Columbia, MO 65211, USA; 4 Department of Plant Biology and Department of Crop Sciences, University of Illinois at Urbana-Champaign, Urbana, IL 61801, USA; 5 W. M. Keck Center for Comparative and Functional Genomics, University of Illinois at Urbana-Champaign, Urbana, IL 61801, USA; 6 Donald Danforth Plant Science Center, St. Louis, Missouri 63132, USA; 7 School of Biotechnology, Yeungnam University, Gyeongsan, Gyeongbuk, 712749 South Korea; 8 Research Support Computing, University of Missouri, Columbia, MO 65211, USA; 9 Bio-Rad Laboratories, 2000 Alfred Nobel Drive, Hercules, CA 94547, USA; 10 School of Biotechnology, Yeungnam University, Gyeongsan, Gyeongbuk, 712749 South Korea; 11 Insightful Corporation, Seattle, WA 98109, USA

## Abstract

**Background:**

Previous work showed that the maize primary root adapts to low Ψ_w _(-1.6 MPa) by maintaining longitudinal expansion in the apical 3 mm (region 1), whereas in the adjacent 4 mm (region 2) longitudinal expansion reaches a maximum in well-watered roots but is progressively inhibited at low Ψ_w_. To identify mechanisms that determine these responses to low Ψ_w_, transcript expression was profiled in these regions of water-stressed and well-watered roots. In addition, comparison between region 2 of water-stressed roots and the zone of growth deceleration in well-watered roots (region 3) distinguished stress-responsive genes in region 2 from those involved in cell maturation.

**Results:**

Responses of gene expression to water stress in regions 1 and 2 were largely distinct. The largest functional categories of differentially expressed transcripts were reactive oxygen species and carbon metabolism in region 1, and membrane transport in region 2. Transcripts controlling sucrose hydrolysis distinguished well-watered and water-stressed states (invertase *vs*. sucrose synthase), and changes in expression of transcripts for starch synthesis indicated further alteration in carbon metabolism under water deficit. A role for inositols in the stress response was suggested, as was control of proline metabolism. Increased expression of transcripts for wall-loosening proteins in region 1, and for elements of ABA and ethylene signaling were also indicated in the response to water deficit.

**Conclusion:**

The analysis indicates that fundamentally different signaling and metabolic response mechanisms are involved in the response to water stress in different regions of the maize primary root elongation zone.

## Background

Water supply limits crop productivity more than any other abiotic factor [1], and the ability of plant roots to find and extract water in drying soil can determine plant reproductive success and survival. Indeed, the adaptation of roots to counteract a limiting water supply is highlighted by the fact that root growth is often less sensitive to water deficit than shoot growth [2,3]. Understanding the mechanisms that allow roots to grow at low water potentials (Ψ_w_) should reveal ways to manipulate drought responses and may ultimately improve tolerance.

Progress in understanding the mechanisms that determine root growth at low Ψ_w _has been made using a maize seedling system involving precise and reproducible imposition of water deficits [4,5]. Root elongation rate under severe water deficit (Ψ_w _of -1.6 MPa) was about 1/3 the rate of growth at high Ψ_w _(-0.03 MPa) [4]. Kinematic analyses detected distinct responses of longitudinal expansion rate to low Ψ_w _in different regions of the root growth zone 48 h after stress imposition when the root elongation rate was at steady state [4,6]. Most striking was the complete maintenance of longitudinal expansion rate in the apical 3-mm region of roots growing at low compared to high Ψ_w_. The adjacent, older, tissue of water-stressed roots decreased expansion rate compared to well-watered roots leading to a shortening of the growth zone.

The biophysical and biochemical bases for the altered growth rate profiles observed in water-stressed roots have been studied (reviewed in [5]). Progressive water deficit induces osmotic adjustment, cell wall loosening, increased ABA accumulation, and membrane hyperpolarization. Little is known about the genes that control these physiologically well documented processes and activities that are involved in the growth response of maize primary roots to severe water deficits. Utilizing the established protocol for stress imposition, we explored the molecular responses to better understand the mechanisms which allowed growth to be maintained in the apical 3-mm but to be inhibited in adjacent older tissues. A maize oligonucleotide microarray was used to identify the differentially expressed transcripts that distinguished well-watered and water-stressed roots in different regions of the root tip in the hopes of delineating the genetic mechanisms responsible for the physiological changes that occur in water-stressed roots and identifying candidate genes that confer the varying growth responses of the different regions of the maize root elongation zone. The results extend some earlier measurements made of gene expression in this system using qRT-PCR by Poroyko et al. [7].

## Results and Discussion

**Figure 1:**
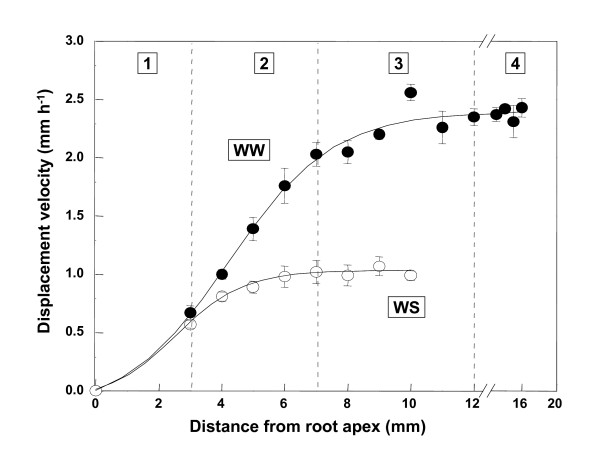
**Displacement velocity as a function of distance from the root cap junction of primary roots of maize (cv FR697) growing in vermiculite under well-watered (WW; Ψ_w _of -0.03 MPa) or water-stressed (WS; Ψ_w _of -1.6 MPa) conditions**. The spatial distribution of longitudinal expansion rate is obtained from the derivative of displacement velocity with respect to position. Regions 1 to 4, as described in the text, are indicated. Reproduced from Sharp et al. (2004) with permission from Oxford University Press.

Kinematic analysis was performed on inbred line FR697 to ensure that the spatial profiles of longitudinal expansion rate in primary roots of seedlings growing at high and low Ψ_w _were similar to those in the hybrid line used in earlier investigations, and, therefore, that FR697 could be used for genetic analysis *in lieu *of the hybrid. Similar to the results with the hybrid, four regions of the root tip with distinctly different elongation characteristics were distinguished (Figure 1; [5]). In water-stressed roots, longitudinal expansion rates were the same as in well-watered roots in the apical 3 mm (region 1), decelerated in the subsequent 4 mm (region 2), and ceased in the following 5 mm (region 3), while in well-watered roots longitudinal expansion rates were maximal in region 2, decelerated in region 3, and did not cease until 12 mm from the apex (region 4).

Three pair-wise comparisons were made of transcripts from water-stressed and well-watered tissues in the different root tip regions. In the first comparison (C1), transcripts from region 1 of water-stressed seedlings were compared with those from region 1 of well-watered seedlings. The second comparison (C2) was made between transcripts from region 2 of the two treatments. We expected a larger number of genes to be differentially expressed in region 2 because its elongation rate decreased greatly under water-stressed compared with well-watered conditions. To prioritize the differentially expressed genes revealed in this comparison, a distinction was made between those genes that are associated with growth inhibition in region 2 specifically as a response to water stress, and those genes that are involved in root cell maturation whether under stress or control conditions. A hypothetical example of the former might be genes involved in auxin response since water stress can increase maize root auxin content [8] and application of exogenous auxin can shorten the root growth zone [9]. An example of the latter might involve genes for secondary wall synthesis [10]. To experimentally make this distinction a third pair-wise comparison (C2/3) was included to compare expression of genes between water-stressed region 2 and well-watered region 3 as these are both regions of growth deceleration. Genes differentially expressed in both C2 and C2/3 are more likely to cause growth inhibition at low Ψ_w _and are not likely to be part of the maturation program itself, whereas genes differentially expressed only in C2 are more likely related to maturation.

**Figure 2:**
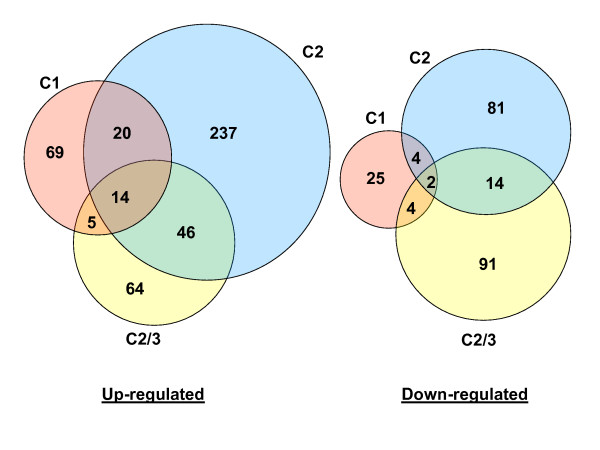
**Venn diagrams illustrating numbers of transcripts up- or down-regulated by water-stress in the three comparisons**. C1 refers to the region 1 comparison, C2 to the region 2 comparison, and C2/3 to the comparison of region 2 of water-stressed roots with region 3 of well-watered roots. All but two transcripts are accounted for in this figure; the other two were up-regulated in one region but down-regulated in another. The three comparisons did not share many of the same differentially expressed transcripts, indicating large differences in the response to water stress between the regions.

**Figure 3:**
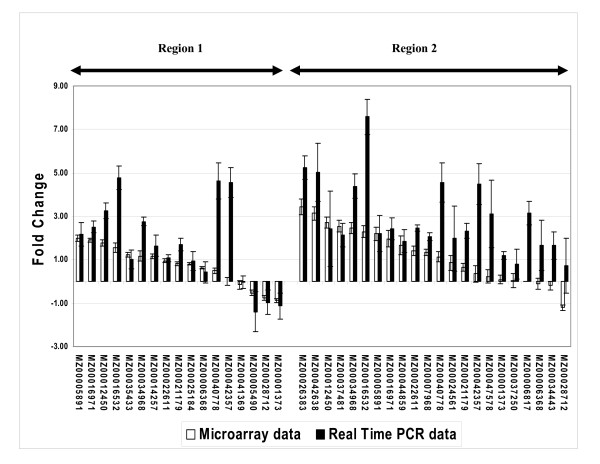
Comparison of real time PCR results with those of the microarray.

An overall view of expression was created for the three comparisons (Figure 2). Using as cutoff the false discovery rate-adjusted P-value of 0.05, 685 differentially expressed transcripts were identified. These represented 678 different ESTs, tentative contigs, or genomic sequences, as indicated in the gal file for the array. The transcripts were divided into either up-regulated (455) or down-regulated (221) categories except for two that changed category between comparisons. The number of affected transcripts was larger in C2 (420) than in C1 (143) (Figure 2), confirming earlier observations based on EST libraries made from these tissues [7]. Comparison of C1 and C2 shows that only a small minority of differentially expressed transcripts were in common: 34 up- and six down-regulated, totaling 7.5% of the 521 transcripts in the two regions. Thus, the response to water stress depended strongly on position within the root elongation zone. There was also only a small overlap between C2 and C2/3: 60 and 16 transcripts were in common between the 386 up- and the 196 down-regulated, respectively. Given our presupposition that only those genes differentially expressed in both C2 and C2/3 are associated specifically with the stress response of region 2, the majority of stress-responsive gene expression was in region 1, the region that adapts to maintain elongation. Accordingly, the majority of differentially expressed transcripts identified in C2 were likely to be involved in root maturation and not specifically in the water stress response: 75% (237/317) of the up-regulated and 80% (81/101) of the down-regulated. Only 16 transcripts were differentially expressed in all three comparisons, underscoring the fact that the response to low Ψ_w _was largely region specific and not dominated by genes that are globally induced by water stress. Real time PCR measurements confirmed the microarray results for all of 17 transcripts studied in region 1 and 22 transcripts studied in region 2 (Figure 3).

Transcripts were divided into three groups according to their expression profiles across the three comparisons. The first group includes those transcripts that might have a primary role in the response of root growth to water stress. Since elongation rates in region 1 were similar in well-watered and water-stressed roots, any differentially expressed transcripts in C1 could have a role in stress adaptation and were placed in the first group regardless of their response in C2 or C2/3. Transcripts differentially expressed in both C2 and C2/3 were also placed in this group. The second group includes those transcripts differentially expressed in C2 alone, which, as explained above, are thought to be part of the root cell maturation program. The third group includes those transcripts whose expression changed only in C2/3 and these were not considered further. While they may be involved in stress response more experiments are needed to interpret their role.

**Figure 4:**
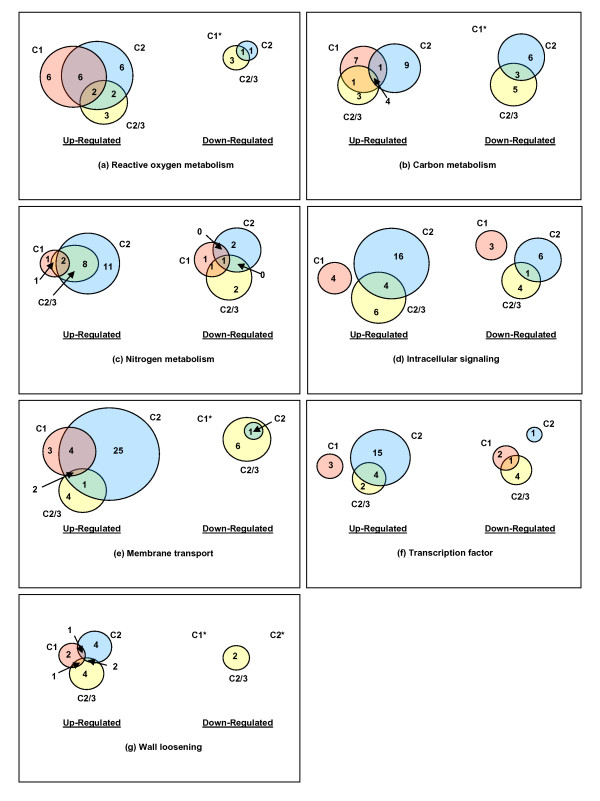
**Regional distribution of expression patterns of water stress-responsive transcripts within specific functional categories**. (a) reactive oxygen species metabolism; (b) carbon metabolism; (c) nitrogen metabolism; (d) intracellular signaling; (e) membrane transport; (f) transcription factors; (g) wall loosening. C1 refers to the region 1 comparison, C2 to the region 2 comparison, and C2/3 to the comparison of region 2 of water-stressed roots with region 3 of well-watered roots. *Denotes regions in which there were no responsive genes in that functional category.

At least 474 of the 678 differentially-expressed transcripts could be annotated and placed into functional categories (Additional file 1). The distribution of expression patterns across functional categories is given in Additional file 2. Of the functional categories identified for transcripts thought to be part of the primary stress response, reactive oxygen species (ROS) metabolism was the largest with 17 transcripts. This was followed by carbon metabolism (16), nitrogen metabolism (12), signaling molecules (12), membrane transport (11), transcription factors (10), and wall-loosening (6) (Figure 4, Additional file 2). In each functional category these transcripts were more often up- rather than down-regulated in water-stressed compared to well-watered roots.

Most differentially expressed transcripts (318) were found in C2 alone and hence are presumed to be involved in the maturation program (Figure 2, Figure 4, Additional files 1 and 2). Membrane transport (25 transcripts) was the functional category with the greatest number and all of these were up-regulated in C2 (Additional file 2). This was followed by signaling molecules (22), transcription factors (16), other DNA-binding proteins (16), carbon metabolism (14), and lipid metabolism (14) (Additional file 2). In each functional category in the maturation program, transcripts were more often up- rather than down-regulated under water stress.

The genes identified here have little in common with those found in an earlier study by Bassani et al. [11] of differentially-expressed genes in different regions of the maize primary root tip under water stress. Only four of the genes found by Bassani et al. had any similarity (evalue < e-10) to transcripts responding in either C1 or C2. The differences in the two studies may be due to growth conditions; Bassani et al. grew plants in the light and imposed a Ψ_w _of -0.5 MPa whereas plants were grown in the dark at -1.6 MPa in our study. Also, Bassani et al. imposed low Ψ_w _using a solution of polyethylene glycol (PEG) which is known to inhibit root growth by limiting oxygen supply in addition to the effects of low Ψ_w _[12].

Differential expression in response to water deficit of a limited set of genes in seminal, lateral, and adventitious root tips was studied in rice by Yang et al. [13,14]. While many of their reported genes had similar function to genes in our study none were orthologous to our gene set. Analysis of gene expression in individual tissues has been performed previously [15] in three longitudinal sections from the apex of well-watered Arabidopsis roots that correspond approximately to the three segments we describe here. Tentative Arabidopsis orthologs (defined in the Methods) to our gene set are reported in Additional file 3.

In what follows selected transcripts from the group of primary stress response genes are first discussed by functional category, followed by consideration of the maturation-related genes, in order to relate their functions to known biochemical and physiological responses to water stress in the maize root tip.

### ROS metabolism

ROS are reactive molecules that can accumulate to toxic levels with water deficit and other stresses. Enzymes that metabolize ROS are therefore important in preventing the damage that excess ROS could cause. Several transcripts for proteins that consume intracellular ROS were up-regulated. A catalase 3 transcript was up-regulated in all three comparisons (MZ00042638) whereas another (MZ00041427) was up-regulated in C1 and C2, confirming results using rtPCR [7], and indicating a need to reduce excess hydrogen peroxide in both regions (Table 1). Several metallothionein-like transcripts were up-regulated in C1 (MZ00039683, MZ00039751, MZ00039699) or in both C1 and C2 (MZ00037083, MZ00013363, MZ00036098). Metallothioneins possess superoxide-and hydroxyl radical-scavenging activities [16]. Thus, at least 11 transcripts were up-regulated whose proteins can decrease peroxide content of the cell interior.

**Table 1:** Selected transcripts involved in ROS metabolism, carbohydrate and proline metabolism, hormone synthesis and hormone response, cell wall loosening proteins, and transport.

**ID**	**C1**	**C2**	**C2/3**	**Annotation**	**Accession ID**	**Evalue**
	Fold Change			**Reactive Oxygen Metabolism**		
MZ00042638	3.8	8.8	5.0	catalase isozyme 3 (EC 1.11.1.6)	gb|AAA33441.1	0
MZ00041427	2.7	4.0		Catalase isozyme 3	sp|P18123	4E-100
MZ00039683	2.3			metallothionein-like protein [Saccharum hybrid cultivar]	gb|AAV50043.1	2E-21
MZ00037083	2.8	12.9		metallothionein- like protein [Zea mays]	emb|CAA57676.1	4E-33
MZ00026815	8.9	3.0		putative oxalate oxidase [Oryza sativa (japonica cultivar-group)]	ref|XP_469352.1	0
MZ00037273	2.0			peroxidase prx15 precursor [Spinacia oleracea]	gb|AAF63027.1	3E-55
MZ00015469		27.2	4.7	putative peroxidase [Oryza sativa (japonica cultivar-group)]	ref|NP_919535.1	0
						
				**Carbon Metabolism**		
MZ00026383	2.9	10.8	3.9	sucrose synthase 3 {Zea mays;}	gb|AAM89473.1	0
MZ00018306		0.3		putative alkaline/neutral invertase {Oryza sativa (japonica cultivar-group);}	gb|BAD33266.1	2E-158
MZ00005490		0.2	0.2	Beta-fructofuranosidase 1 precursor (EC 3.2.1.26) {Zea mays;}	sp|P49175	1E-46
MZ00014257	2.2			Glucose-1-phosphate adenylyltransferase large subunit 2 (EC 2.7.7.27)	sp|P55234	1E-264
MZ00021179	1.8			Putative starch synthase {Oryza sativa (japonica cultivar-group);}	gb|AAK98690.1	8E-17
MZ00041252	2.2			myo-inositol 1-phosphate synthase {Zea mays;}	gb|AAG40328.1	8E-271
MZ00015192		0.1	0.1	putative myo-inositol oxygenase {Oryza sativa (japonica cultivar-group);}	gb|BAD53821.1	5E-152
MZ00025596	3.5	5.2	4.0	putative delta l pyrroline-5-carboxylate synthetase {Oryza sativa}	gb|BAB64280.1	8E-209
MZ00027872	0.2	0.1	0.1	putative proline oxidase {Oryza sativa (japonica cultivar-group);}	gb|AAP54933.1	3E-150
						
				**Hormones**		
MZ00051675	0.5			CIPK-like protein {Oryza sativa (japonica cultivar-group);}	gb|AAP82174.1	1E-40
MZ00019036		3.0	2.2	putative protein phosphatase 2C {Oryza sativa (japonica cultivar-group);}	gb|AAT58680.1	3E-155
MZ00028000		3.6		putative protein phosphatase 2C {Oryza sativa (japonica cultivar-group);}	gb|AAT58680.1	2E-87
MZ00016125		3.0	3.1	protein phosphatase 2C-like protein {Oryza sativa (japonica cultivar-group);}	gb|BAC05575.1	2E-162
MZ00007968		2.5	2.5	TRAB1 [Oryza sativa (japonica cultivar-group)]	ref|XP_482899.1	3E-17
MZ00051037	1.6			ABF3 (ABSCISIC ACID RESPONSIVE ELEMENTS-BINDING FACTOR 3)	ref|NP_567949.1	3E-14
MZ00026642	4.2		6.1	dehydrin [Zea mays]	gb|AAA33480.1	0
MZ00041440		5.2		dehydrin [Zea mays]	gb|AAA33480.1	0
MZ00042357a	4.5	4.5		Group 3 Lea protein MGL3 [Zea mays]	emb|CAA82632.1	3E-76
MZ00015996	1.2			putative Ubiquitin ligase SINAT5 [Oryza sativa (japonica cultivar-group)]	ref|XP_465055.1	0
MZ00035785	1.8			jacalin homolog [Oryza sativa (japonica cultivar-group)]	gb|ABA97248.1	2E-15
MZ00024083	12.4			JI23_HORVU 23 kDa jasmonate-induced protein	sp|P32024	2E-21
MZ00050071	0.5			ethylene-binding protein-like [Oryza sativa (japonica cultivar-group)]	dbj|BAD38371.1	2E-64
						
				**Wall Loosening**		
MZ00021464		2.2		putative endoxyloglucan transferase [Oryza sativa]	ref|NP_922874.1	2E-77
MZ00016971	3.7			alpha-expansin 1 [Zea mays]	gb|AAK56119.1	0
MZ00030567	2.4			alpha-expansin [Oryza sativa (japonica cultivar-group)]	ref|XP_475418.1	0
MZ00029301		8.0	6.3	beta-expansin [Oryza sativa]	gb|AAF72988.1	0
MZ00036823	1.9		1.9	putative endo-1,3;1,4-beta-D-glucanase [Oryza sativa (japonica cultivar-group)]	gb|AAU10802.1	8E-17
						
				**Transport**		
MZ00025001	4.6	12.1	5.7	Putative anion transporter [Oryza sativa]	ref|XP_470223.1	0
MZ00006817	3.0			putative ripening regulated protein [Oryza sativa (japonica cultivar-group)]	dbj|BAD46507.1	7E-36
MZ00011868	2.2			putative transmembrane protein [Oryza sativa (japonica cultivar-group)]	ref|NP_920876.1	2E-22
MZ00012450	3.4	6.5		putative amino acid transport protein [Oryza sativa (japonica cultivar-group)]	ref|XP_463772.1	3E-56
MZ00043256	1.5	6.5		sorbitol transporter [Malus × domestica]	dbj|BAD42344.1	1E-29
MZ00031622	1.5			oligopeptide transporter OPT-like [Oryza sativa (japonica cultivar-group)]	ref|XP_466910.1	1E-80
MZ00001869		0.4	0.5	putative organic cation transporter [Oryza sativa (japonica cultivar-group)]	ref|XP_478718.1	0

Legend. C1 refers to the region 1 comparison, C2 to the region 2 comparison, and C2/3 to the comparison of region 2 of water-stressed roots with region 3 of well-watered roots.

Some amount of ROS production may be required for growth, however. For example, apoplastic ROS [17] and the enzymes that produce them [e.g., [18,19]] have been implicated in growth control via cell wall loosening. Increased abundances of oxalate oxidase and peroxidase proteins, and increased levels of ROS, have been detected in the apoplast of region 1 of the maize primary root under water-stressed conditions [20]. The increased expression by water stress of putative oxalate oxidase transcripts (MZ00026815) in C1 and C2 may thus be involved in regulation of cell expansion. Enhanced apoplastic peroxide content was reported in transgenic maize over-expressing a wheat oxalate oxidase [21], although how the transgene affected growth in the root tip was not described. Over-expression of class III peroxidases in rice caused increased elongation of the root and root cortical cells presumably by generating peroxide [19]. It is unknown whether the up-regulated transcripts for class III peroxidases in C1 (MZ00037273) and in C2 and C2/3 (MZ00015469) also stimulate growth.

### Carbon metabolism

Control of carbohydrate flow to the root tip is determined in part by the sucrose-hydrolyzing enzymes invertase and sucrose synthase. Two distinct invertase transcripts (MZ00005490, MZ00018306) were down-regulated in C2 whereas a sucrose synthase 3 (SUSY3) transcript (MZ00026383) was up-regulated in C1 and C2. Another SUSY3 transcript (MZ00040720) was also up-regulated in C2. SUSY3 was discovered in maize kernels deficient in the two other known sucrose synthases (SH1 and SUS1) [22], and this is the first indication of a role for this gene outside of the kernel and in a stress response. An advantage in ATP consumption, phosphorous use efficiency, and in the creation of sink strength is provided by employing sucrose synthase over invertase in sucrose metabolism [23].

Glucose-1-phosphate (G1P) is a product of SUSY3 and is a substrate for ADP-glucose pyrophosphorylase (ADGase), the first committed step in starch synthesis. Transcripts for the large subunit of ADGase (MZ00014257) and for a putative starch synthase (MZ00021179) were both up-regulated in C1 alone, suggesting increased starch synthesis which might promote carbon flow to the root tip. The tentatively orthologous rice transcript (Genbank accession: AK100910) to this ADGase increased expression in response to the combination of ABA and sugar [24]. ABA also can greatly enhance the induction by sugar of the large subunit of ADGase in Arabidopsis [25]. Birnbaum et al. [15] reported that the tentative Arabidopsis ortholog is most expressed in all tissues studied nearest the apex of the root (Additional file 3).

Transcripts coding for two activities that regulate inositol contents were differentially expressed in both C1 and C2. Transcripts for *myo*-inositol-1-phosphate synthase (MIPS) (MZ00041252, MZ00038878), which synthesizes *myo*-inositol, was up-regulated in C1 exclusively whereas transcripts for *myo*-inositol oxygenase (MZ00015192, MZ00015195), which catabolizes *myo*-inositol, were down-regulated in C2 and in C2/3. Taken together, these results suggest a stress-induced increase in *myo*-inositol content which could be used for (1) conjugation of auxin, (2) as a compatible solute by itself or as a methyl ether, (3) in membrane lipid synthesis, (4) in raffinose synthesis, (5) in UDP-sugar synthesis, and (6) in phytate and phosphoinositide synthesis [26].

### Nitrogen metabolism

Transcripts for a putative δ-1-pyrroline-5-carboxylate (P5C) synthetase (e.g., MZ00025596), which catalyzes the rate-limiting step in proline synthesis, were up-regulated in all three comparisons (Table 1). Transcripts for a putative proline oxidase (e.g., MZ00027872) were down-regulated in all three comparisons (Table 1). Since altered metabolism in the root tip was not the main cause of proline accumulation with water stress [27], these changes in expression likely act only to supplement the proline pool.

### Hormones

The accumulation of high concentrations of ABA is required for the maintenance of elongation in water-stressed maize roots [28-30], although these same high concentrations of ABA inhibit root growth at high Ψ_w _[30,31]. Thus, the growth-inhibiting ability of ABA must be diminished at low Ψ_w _while permitting the growth-maintaining functions of ABA to operate. Accordingly, we hypothesized that some components of the ABA response are attenuated by stress while others are not.

Transcripts differentially expressed at low Ψ_w _which may be part of the mechanism of ABA action in maize root tips fell into three categories: (a) protein kinases, (b) protein phosphatase type 2C (PP2C) proteins, and (c) transcription factors.

(a) A transcript (MZ00051675) for a CIPK3-like protein was down-regulated by stress in C1 alone (Table 1). CIPK3 is a ser/thr protein kinase involved with calcium sensing in the ABA- and stress- responses of Arabidopsis [32], suggesting this part of the ABA-signaling pathway might be suppressed in maize roots growing at low Ψ_w_.

(b) Three transcripts for protein phosphatase-like proteins known to restrict ABA response in Arabidopsis roots and other tissues were up-regulated in C2 (ABI1-like; MZ00028000) or also in C2/3 (PP2C-HAB1, MZ00019036; PP2C-HAB2, MZ00016125) (Table 1). In Arabidopsis, PP2C-HAB1 [33], PP2C-HAB2 [34], and ABI1 [35] each act as negative regulators of ABA response, and so perhaps attenuate root response to ABA under water stress.

(c) Two transcripts for bZIP family transcription factors were up-regulated by stress. The first (MZ00007968) represents TRAB1, a transcription factor that interacts with the OSVP1 protein to induce gene expression in rice [36], which increased in C2 and in C2/3. Rice TRAB1 is expressed in roots and is inducible by ABA [36].

The second transcript is for an Arabidopsis ABA-response element-binding protein (ABF3) (MZ00051037), which exhibited increased expression in C1. Rice plants over-expressing OsDREB1a, a rice homolog of ABF3, displayed retarded growth and increased proline and sugar content when grown under normal conditions. They also demonstrated improved recovery from water deprivation [37].

Some potentially ABA-inducible transcripts were already mentioned. In addition, a maize dehydrin up-regulated in C1 and C2/3 (MZ00026642) and a second up-regulated in C2 alone (MZ00041440) were tentative orthologs of the rice LIP9 dehydrin. LIP9 was up-regulated in the OsDREB1a over-expressing plants mentioned above [36] and in response to ABA and drought in rice [38]. Dehydrins are expected to help protect cells from stress.

Water-stress can increase auxin levels in maize root tips [8] and exogenous auxin can shorten the elongation zone while promoting growth in the apical region of cereal roots [9]. This suggests that auxin may play a role in root growth at low Ψ_w_. A transcript (MZ00015996) for a putative SINAT5, a ubiquitin protein ligase, was up-regulated by stress in C1. *SINAT5 *expression is enhanced by auxin in root tips of Arabidopsis [38] and increased expression of SINAT5 protein in transgenic Arabidopsis promoted root elongation [39]. Thus, the *SINAT5*-like gene product may act to maintain cell elongation in region 1 of water-stressed maize primary roots.

The up-regulation in C1 of a transcript similar to a 23-kD jasmonate-induced thionin (MZ00024083) suggests some action of jasmonates due to stress. Thionins are involved in plant defenses to biotic factors [40]. Jasmonates are also able to induce some genes of the jacalin family of lectins which are associated with defense responses. A transcript for a jacalin-like protein was up-regulated in C1 (MZ00035785).

In previous studies, some of the response to endogenous ABA in roots at low Ψ_w _was attributed to its ability to prevent synthesis of excess ethylene, which otherwise would inhibit root elongation and promote radial swelling [41]. A transcript (MZ00050071) for an ethylene-binding-like protein was down-regulated in C1. Reduced ability to bind ethylene should make the root less sensitive to ethylene, perhaps influencing root shape. It is noteworthy that maize primary roots are thinner at low compared to high Ψ_w _[4,6].

### Wall loosening proteins

The increased wall extensibility in region 1 of water-stressed roots [42] may be due to increased activity of cell wall loosening proteins. Increased activity of xyloglucan endotransglycosylase (XET) was reported in region 1 of water-stressed roots, and was shown to be ABA-dependent [43]. A transcript for XET (MZ00021464) was up-regulated in C2 (Table 1) but not in C1 where the enzyme activity increases [43]. This suggests that the increased enzyme activity in region 1 was due to post-transcriptional events.

Expansins are also associated with increased wall-loosening in water-stressed maize root tips [42]. Two transcripts for α-expansins (*exp1*, MZ00016971; *exp5*, MZ00030567) were up-regulated in C1, while β-expansins (e.g., *expB3*, MZ00029301) were up-regulated in C2 and C2/3. These data confirm previous measures of increased expression of α-expansin genes and *expB6 *in stressed maize root tips [44]. It is unclear what role β-expansins play in the regulation of growth in region 2 at low Ψ_w_, in which elongation was inhibited, as they are able to loosen walls [45].

The major hemicellulose class of the maize primary cell wall is composed of mixed linkage β-glucans which are believed to be cleaved by endo-1,3;1,4-beta-D-glucanases to cause wall loosening [46]. A transcript for a putative endo-1,3;1,4-beta-D-glucanase was up-regulated in C1, and an endo-1,3;1,4-beta-D-glucanase was identified in the maize primary root elongation zone in a cell wall proteomic study of well-watered roots [47]. More recently, however, a comprehensive study on root region specific cell wall protein profiles showed decreased abundance of two endo-1,3;1,4-beta-D-glucanases in region 1 under water deficit conditions [20]. These observations suggest that changes at the transcript level for this particular member may not be reflected at the translational level, or that members of this gene family may have different subcellular localizations [48].

### Membrane transport

Ober and Sharp [49] reported that maize root tip cortical cell membranes are hyperpolarized by stress and that the hyperpolarization requires increased H^+^-ATPase activity of the plasma membrane. Potassium and chloride ions are also important for the hyperpolarization. When ABA is prevented from accumulating the membrane becomes more hyperpolarized in the apical 2- to 3-mm, suggesting that ABA acts on ion transport or transporters in the regulation of growth. We hypothesized that changes in expression of genes for such transporters occur in this region. Two putative anion transporters were up-regulated in all three comparisons (MZ00025001, MZ00043643) and a third in C1 and C2 (MZ00009288) which might serve this function (Table 1).

Two transcripts coding for proteins with similarity to MATE efflux family proteins were increased in C1 (MZ00006817, MZ00011868) and a third in both C1 and C2 (MZ00030937). The functions of only a few MATE proteins are known [50,51] although some respond to phosphate- [52] or iron-deficiency [53], conditions which may accompany water stress. A transcript for a putative amino acid transporter (MZ00012450) was up-regulated in C1 and C2 as was one for a sugar transport family protein (MZ00043256), possibly in response to enhanced nutritional requirements. A transcript for an oligopeptide transporter-like gene (MZ00031622) was increased in C1, although no functional characterization is available [54].

### Root maturation-related genes

Transcripts were indentified that were presumed to be related to tissue maturation in region 2 of stressed roots and in region 3 of control roots and not directly responsive to water stress. Such genes might function in cell-wall thickening, vascular differentiation, and increased resistance to water and solute transport, among other processes. Some pertinent transcripts are listed in Table 2.

**Table 2:** Selected transcripts which are likely to be involved in cell wall maturation regardless of water status.

**ID**	**C1**	**C1**	**C2/3**	**Annotation**	**Accession ID**	**Evalue**
	Fold Change			**Root Maturation**		
MZ00008104		3.1		ABC transporter family protein-like {Oryza sativa (japonica cultivar-group);}	gb|BAC84400.1	1E-13
MZ00018690		2.4		gibberellin 20-oxidase 1 [Lolium perenne]	gb|AAY67841.1	0
MZ00007636		2.1		Gibberellin 2-oxidase [Oryza sativa (japonica cultivar-group)]	ref|XP_475621.1	1E-19
MZ00050533		2.2		mechanosensitive ion channel domain-containing protein-like {Oryza}	gb|BAD28130.1	5E-117
MZ00016581		5.4		NOD26-like membrane integral protein ZmNIP2-1 {Zea mays;}	gb|AAK26751.1	6E-143
MZ00026069		1.9		O-methyltransferase {Secale cereale;}	gb|AAO23335.1	4E-114
MZ00004720		22.9		O-methyltransferase ZRP4 (EC 2.1.1.-) (OMT). {Zea mays;}	sp|P47917	7E-69
MZ00034353		2.6		phytase {Zea mays;}	gb|CAA11391.1	1E-199
MZ00026517		4.0		putative gibberellin regulated protein [Oryza sativa (japonica cultivar-group)]	gb|AAR87222.1	4E-32
MZ00046781		3.3		putative ABC transporter protein {Arabidopsis thaliana;}	gb|AAK92745.1	2E-85
MZ00049827		2.7		putative amino acid transport protein {Oryza sativa (japonica cultivar-group);}	gb|BAD08181.1	2E-86
MZ00044334		3.3		putative amino acid transporter {Oryza sativa (japonica cultivar-group);}	gb|AAV24773.1	2E-149
MZ00012753		5.0		putative inositol polyphosphate 5-phosphatase [Oryza sativa]	ref|XP_550422.1	3E-47
MZ00041660		7.0		putative MATE efflux family protein {Oryza sativa (japonica cultivar-group);}	gb|AAS01970.1	2E-74
MZ00019635		3.9		putative multidrug resistance p-glycoprotein {Oryza sativa (japonica cultivar-group);}	gb|BAD16475.1	4E-66
MZ00019481		4.4		putative nitrite transporter {Oryza sativa (japonica cultivar-group);}	gb|BAD54372.1	1E-20
MZ00025206		8.2		putative o-methyltransferase ZRP4 {Oryza sativa (japonica cultivar-group);}	gb|AAP51889.1	7E-72
MZ00005402		1.8		putative PDR-like ABC transporter {Oryza sativa (japonica cultivar-group);}	gb|BAD53546.1	2E-74
MZ00028553		2.9		putative phytase {Oryza sativa (japonica cultivar-group);}	gb|AAO73273.1	1E-248
MZ00052125		3.1		putative proton-dependent oligopeptide transporter (POT) {Oryza}	gb|AAT85250.1	4E-120
MZ00048363		2.3		putative sialin {Oryza sativa (japonica cultivar-group);}	gb|BAD46232.1	4E-87
MZ00021212		3.2		putative sugar transporter {Oryza sativa (japonica cultivar-group);}	gb|BAD21843.1	5E-52
MZ00026965		4.4		Putative sulfate transporter {Oryza sativa (japonica cultivar-group);}	gb|AAN59769.1	3E-127
MZ00048706		4.0		Putative sulfate transporter ATST1 {Oryza sativa (japonica cultivar-group);}	gb|AAN06871.1	3E-102
MZ00044209		3.2		putative Zn and Cd transporter {Thlaspi caerulescens;}	gb|CAC86389.1	1E-19
MZ00041461		2.8		Triose phosphate/phosphate translocator, chloroplast precursor (CTPT). {Zea mays;}	sp|P49133	3E-213

Legend. C1 refers to the region 1 comparison, C2 to the region 2 comparison, and C2/3 to the comparison of region 2 of water-stressed roots with region 3 of well-watered roots.

Inositol phosphates such as inositol 1,4,5-triphosphate (IP_3_) [55] and inositol hexakisphosphate (IP_6_, or phytate) [56] have roles in intracellular signaling. Inositol 5-phosphatase can decrease content of IP_3 _and in Arabidopsis it is induced by ABA [57]. Phytase dephosphorylates phytate. Phytate is synthesized in maize roots [58] and phytase mRNA and protein have been localized in the pericycle, endodermis, and rhizodermis of maize root tips [59]. Transcripts for enzymes that could metabolize inositol phosphates, one for inositol 5-phosphatase (MZ00012753) and two for phytase (MZ00034353, MZ00028553), were up-regulated by stress in C2. Little is known about the role of inositol phosphate signaling in root development or its response to water stress.

Poroyko et al. [7] found that transcripts for inorganic ion and water transport and metabolism were generally up-regulated in region 2. We found some 25 transcripts whose functions are related to membrane transport were up-regulated in C2 alone. Cells in the more mature region of the expanding root tip have decreased symplastic continuity with the phloem [60]. As a consequence solutes and water must traverse more membranes to be taken up by cells. Many of these transporters may be part of that response. For example, it is expected that increased uptake from the apoplast of sugars and amino acids is required, and consistent with this idea several putative sugar and amino acid transporters were up-regulated. The differential regulation of several sulfate transporters was notable since sulfate content increases in the xylem of more mature maize plants of this genotype under water stress conditions [61]. Transcripts for ABC transporters were identified as well, belonging to the EPD family that is not yet well described in plants [62].

Expression increased in C2 alone for three O-methyl transferase transcripts (MZ00004720, MZ00026069, MZ00025206). These may be involved in creating phenylpropanoid precursors to lignin and suberin whose contents increase in mature roots [63].

Up-regulated transcripts for GA metabolism (MZ00007636, gibberellin 2-oxidase; MZ00018690, gibberellin 20-oxidase) and response (MZ00026517, putative gibberellin regulated protein) were identified in C2. The Arabidopsis tentative ortholog was also most expressed in tissues of this region of the root apex (Additional File 3; [15]). A role for GA in root cell growth was previously indicated by the altered pattern of radial swelling observed in GA-deficient maize seedlings [64].

### Promoter analysis

The regulatory mechanisms of genes are mostly controlled by the binding of transcription factors to the sites located upstream of coding regions. Possible transcription factor binding sites (*cis *elements) of the differentially expressed genes found in this study were sought. Promoter regions were defined as the 1,000 bases upstream of the coding regions of full sequence gene models for maize (available from The TIGR Maize Database), or for tentatively orthologous rice and Arabidopsis genes. *Cis *elements were identified in the promoters of 167 maize genes or their tentative orthologs using the PLACE database. While 61 classes of cis elements were detected (Additional file 4) there was little difference in their distribution between sequences that belonged to the primary or maturation classes of transcripts, and hierarchical clustering techniques did not reveal any associations with specific expression patterns (not shown).

## Conclusion

We explored gene expression in the maize primary root to identify causes for the changes observed in the spatial pattern of root elongation at low Ψ_w_. The two regions of the root studied showed distinctly different transcript profiles underscoring the importance of spatial analysis. Within region 1, where longitudinal expansion rate is maintained during stress, all differentially expressed transcripts were considered to be part of the mechanism of adaptation to stress. Within region 2, the region where longitudinal expansion decreases from the maximal control rate to a progressively slower rate under stress, transcripts were divided into two groups: those that were part of the stress response that brought about early root cell maturation, and those that were part of maturation itself. Region 1 contained a greater number of differentially expressed genes involved in the stress response than did region 2, even though region 2 had the greater total number of differentially expressed genes. This result was expected given the maintenance of elongation in region 1 and its inhibition in region 2 of water-stressed roots.

Our results support and add molecular details to the model of root growth maintenance under stress via increased wall loosening in region 1, osmotic adjustment, regulation by ABA, and changes in membrane transport. The data suggest a need for control of intracellular ROS content by catalase and metallothioneins and for apoplastic hydrogen peroxide production by oxalate oxidase and other cell wall proteins to cause wall loosening. A transcript with similarity to a mixed linkage β-glucanase suggests a role for this enzyme in stress adaptation in the root growth zone. Carbohydrate metabolism appears altered at the transcript level to involve roles for SUSY3 and enhanced starch synthesis. The mechanism of osmotic adjustment by proline accumulation was extended to include changes in expression of genes for proline metabolism. Altered expression of transcripts similar to known members of the ABA signaling pathway suggest some parts of the ABA response network are attenuated while others are not, which may explain how the stressed root tolerates, and requires, high endogenous levels of this hormone. The stress-enhanced expression of a *SINAT5*-like transcript may link auxin to growth maintenance in region 1. Change in an ethylene-binding like protein is suggested to help control the shape of the stressed root. Evidence for jasmonate-induced gene expression was also indicated that is probably related to biotic stress defense. The up-regulated transcripts for membrane anion transport may bring about the known stress-induced changes in membrane potential. Together the data show that the regulation of root growth at low water potentials involves region-specific changes in many different aspects of cell metabolism, signaling, and transport.

## Methods

### Maize seedling culture and root harvest

Maize (*Zea mays *L. cv FR697) seeds were imbibed for 24 h in 1 mM CaSO_4_. Seeds were then germinated for 28 h in vermiculite well-moistened with 1 mM CaSO_4 _at 29°C in the dark [41]. Seedlings with primary roots 12–20 mm in length were transplanted into vermiculite mixed with pre-determined amounts of 1 mM CaSO_4 _to create high (-0.03 MPa) or low (-1.6 MPa) Ψ_w _and grown under near-saturating humidity conditions to prevent further drying of the media. Vermiculite Ψ_w_was measured by isopiestic thermocouple psychrometry [65].

By combining harvests from a series of experiments, four biological replicates of 440 pooled well-watered and 660 pooled water-stressed primary roots were collected at 48 h after transplanting (using a green safelight; [29]). The apical 12 mm of each root was sectioned into three regions based on previously-characterized longitudinal expansion rate profiles (Figure 1; distances are from the junction of the root apex and root cap): region 1, 0–3 mm plus the root cap; region 2, 3–7 mm; region 3, 7–12 mm. Samples were collected by position and immediately frozen in liquid nitrogen.

### RNA isolation

Total RNA was isolated from maize root apical segments using Trizol reagent following the manufacturer's instructions (Invitrogen Corp., Carlsbad, CA). Residual DNA was removed by *Dnase *I (Invitrogen, Carlsbad, CA) treatment for 15 min at room temperature, followed by use of RNeasy columns (Qiagen, Valencia, CA).

### Microarray, hybridization, and data analysis

Gene expression changes were assessed using pair-wise comparisons of water-stressed region 1 with well-watered region 1 (designated C1), water-stressed region 2 with well-watered region 2 (designated C2), and water-stressed region 2 with well-watered region 3 (designated C2/3). Maize oligonucleotide arrays printed at the University of Arizona were used [66]. Each maize array consisted of two slides that together contained 57,452 unique oligos, mostly 70-mers. The Maize Root Genomics Project [67] contributed 668 novel sequences to the array. Overall, 30,000 genes were represented on the array. Conservative estimates place the maize transcriptome at 59,000 genes ([68]; H Bohnert, unpublished). More details about the array can be found in Gardiner et al., [69]. Additional annotation of the parent sequences to the oligos was performed by blastx search of protein databases (NR) at NCBI [70], UniProt [71], or TAIR [72]. First strand cDNAs were synthesized from 50 μg of total RNA using anchored oligo(dT)24 primers with SuperScript III RT (Invitrogen, Carlsbad, CA), and aminoallyl-dUTP was incorporated into the cDNAs. The RNA template was removed by treatment with RnaseH (Invitrogen, Carlsbad, CA), and cDNAs were purified to remove unincorporated aminoallyl-dUTP using Microcon 30 spin concentrators (Millipore Corp., Bedford, MA). Following purification, monoreactive-Cye5 or Cye3 dyes (Amersham Biosciences Corp., Piscataway, NJ) were conjugated to aminoallyl-dUTP on the cDNAs and the unconjugated dye was removed using Qiagen PCR purification columns. The purified Cy3 and Cy5-labeled cDNAs were concentrated to 60 μl and hybridized to the maize oligonucleotide array for 16–18 h at 42°C. Following hybridization, the arrays were washed three times, twice with medium stringency buffer (1× SSC, 0.2% SDS) and once with high stringency buffer (0.1× SSC, 0.2% SDS). Washed slides were dried and scanned immediately using a GenePix scanner (GenePix^® ^4000B, Axon Instruments, Inc.) at 532 nm (17 mW) and 635 nm (10 mW). GenePix Pro 4.1 software was then used to extract spot intensity data.

Each of the three comparisons included 16 slides corresponding to four biological replications of two slides each with dye-swap. The R programming environment, including the limma package, was used to process and statistically analyze the data. Mean foreground intensity values were log transformed and subjected to lowess normalization to correct for intensity-dependant dye effects. To obtain accurate and precise estimates of gene expression values a mixed linear model was applied which was based on a two-step approach essentially as described by Wolfinger et al. [73].

The mixed linear model that was fit across genes is

*y*_*ijklg *_= μ + *T*_*i *_+ *D*_*j *_*+ *(*TD*)_*ij *_*+ R*_*k *_+ (*A/TD*)_*ijl *_*+ *(*A/R*)_*kl *_*+ e*_*ijklg*_

where *y*_*ijklg *_is the log intensity value for the *g*^*th *^gene with treatment *i*, dye *j*, and replicate *k *on the *l*^*th *^array, μ is the overall mean across all factors, *T*_*i *_is the overall effect of treatment *i*, *D*_*j *_is the overall effect of dye *j*, *R*_*k *_is overall effect of replicates, (*TD*)_*ij *_is the interaction of the *i*^*th *^treatment and *j*^*th *^dye, (*A/TD*)_*ijl *_is the effect of *l*^*th *^array within *i*^*th *^treatment and *j*^*th *^dye, (*A/R*)_*kl *_is the effect of *l*^*th *^array within *k*^*th *^replicate, and *e*_*ijklg *_is the residual error term, i.e. variation that is not explained by the factors included in the model. In the model the treatment and the dye effects were treated as fixed, and the replicate and the array effects within replicate or treatment by dye effect as random. Residuals obtained from the global model were fit, one gene at a time, to the following mixed model:

*r*_*ijklg *_= *μ *+ *T*_*i *_+ *D*_*j *_+ *R*_*k *_+ (*A/R*)_*kl *_+ *e*_*ijklg*_

where the effects fit in the model were treated the same as in the global model. False discovery rate (FDR) adjusted *P*-values were determined for 64,870 spots in C1, 56,609 spots in C2 and in C2/3. The difference in numbers of spots was due to the removal from the second and third sets of all values with saturated intensities. The threshold for the FDR was set at 0.05, i.e., there is a 5% chance that the designation of significance is false.

We define as "tentatively orthologous" a sequence from another species if it was the top scoring match in both parts of a reciprocal BLAST analysis pitting the entire set of maize array sequences with all known genes of that species as defined by TAIR (Arabidopsis) or TIGR (rice).

### Promoter Analysis

Promoter regions were deemed to be the 1,000 bases upstream of the coding regions of the maize sequence full gene models available from The TIGR Maize Database (AZM version 5 [74]). Similar promoter sequences were obtained for tentatively orthologous rice genes from TIGR Rice Genome Annotation [75] and Arabidopsis [72]. Motifs listed in the PLACE database [76] were identified in each of the three sets of promoters using the PLACE website. PLACE was constructed and maintained at the National Institute of Agrobiological Sciences (NIAS) and was made available without charge.

### Verification of microarray data by gene specific relative quantitative RT-PCR

To validate the differential expression pattern obtained from the microarray analysis, transcripts from the same RNA samples of well-watered and water-stressed region 1 and region 2 tissues were quantified using real-time PCR. cDNA was synthesized according to the Taqman RT kit protocol (ABI, Foster City, CA). PCR primers were designed using Primer Express 2.0 (Applied Biosystems, ABI) to create amplicons of 100 to 150 bp. The experiment was performed for three biological replicates using the *Zea mays *actin gene (gi|21206665) as an endogenous control. The real-time measurements were carried out with the GeneAmp 7000 Sequence Detection System (Applied Biosystem) using the standard protocol.

## Authors' contributions

WGS, WT, BV and KC participated in the design of experiments, microarray hybridization, microarray analysis, qRT-PCR validation of array results, bioinformatic analysis, data interpretation and manuscript preparation. LGH, MEL and JZ conducted the physiology experiments and collected the root tissues. J-JK and DH helped in the statistical analysis of microarray data. HJB, DPS, GED, GKS, RES and HTN participated in the design of experiments, data interpretation and revision of the manuscript. All authors read and approved the final manuscript.

## Supplementary Material

Additional file 1 List of differentially expressed transcripts by their Operon ids and their relationship to the most similar translation product in NR. Genes are categorized according to primary stress response, maturation related response and functional classification. The parent est sequence identifier, the fold change, the FDR-adjusted p value, and the accession ID, annotation, score, and evalue obtained by BLASTX alignment against the non-redundant protein database at NCBI are given. Click here for file

Additional file 2 Functional categories and patterns of differential expression across the three comparisons. Click here for file

Additional file 3 Tentatively orthologous Arabidopsis genes and a description of their expression profile from Birnbaum et al. [15] Click here for file

Additional file 4 Distribution of *cis *elements found in the region up to 1,000 bases upstream of the coding sequences of maize genes identified in this study, or in tentative orthologs of genes identified in this study. Click here for file
